# Development of Inclusion Complexes With Relative Humidity Responsive Capacity as Novel Antifungal Agents for Active Food Packaging

**DOI:** 10.3389/fnut.2021.799779

**Published:** 2022-01-04

**Authors:** Cristina Muñoz-Shugulí, Francisco Rodríguez-Mercado, Carolina Mascayano, Andrea Herrera, Julio E. Bruna, Abel Guarda, María J. Galotto

**Affiliations:** ^1^Packaging Innovation Center (LABEN), University of Santiago of Chile (USACH), Santiago, Chile; ^2^Center for the Development of Nanoscience and Nanotechnology (CEDENNA), University of Santiago of Chile (USACH), Santiago, Chile; ^3^Department of Food Science and Technology, Technological Faculty, University of Santiago of Chile (USACH), Santiago, Chile; ^4^Department of Environmental Sciences, Faculty of Chemistry and Biology, University of Santiago of Chile (USACH), Santiago, Chile

**Keywords:** allyl isothiocyanate, active packaging, beta-cyclodextrin, *Botrytis cinerea*, inclusion complexes, gas chromatography, headspace, responsive release

## Abstract

**Background:** Allyl isothiocyanate is an excellent antimicrobial compound that has been applied in the development of active food packaging materials in the last years. However, the high volatility of this compound could prevent a lasting effect over time. In order to avoid this problem, cyclodextrin inclusion complexes have been proposed as an alternative, being beta-cyclodextrin (β-CD) as the main candidate. In addition, β-CD could act as a relative humidity-responsive nanoparticle. In this regard, the aim of this study was to develop inclusion complexes based on β-CD and AITC as relative humidity-responsive agents, which can be used in the design of active food packaging materials.

**Methods:** Two different β-CD:AITC inclusion complexes (2:1 and 1:1 molar ratios) were obtained by the co-precipitation method. Entrapment efficiency was determined by gas chromatography, while inclusion complexes were characterized through thermal, structural, and physicochemical techniques. Antifungal capacity of inclusion complexes was determined in a headspace system. Furthermore, the AITC release from inclusion complexes to headspace at different percentages of relative humidity was evaluated by gas chromatography, and this behavior was related with molecular dynamic studies.

**Key Findings and Conclusions:** The entrapment efficiency of inclusion complexes was over to 60%. Two coexisting structures were proposed for inclusion complexes through spectroscopic analyses and molecular dynamic simulation. The water sorption capacity of inclusion complexes depended on relative humidity, and they exhibited a strong fungicide activity against *Botrytis cinerea*. Furthermore, the AITC release to headspace occurred in three stages, which were related with changes in β-CD conformational structure by water sorption and the presence of the different coexisting structures. In addition, a strong influence of relative humidity on AITC release was evidenced. These findings demonstrate that β-CD:AITC inclusion complexes could be used as potential antifungal agents for the design of food packaging materials, whose activity would be able to respond to relative humidity changes.

## Introduction

Allyl isothiocyanate (3-Isothiocyanatoprop-1-ene, AITC) is an oily organosulfur compound with an aliphatic structure produced by Brassicaceae family (1). It is the major antimicrobial component in the essential oils from black (*Brassica nigra*) and brown mustard (*Brassica juncea*), and it is approved as GRAS by the FDA (2). Vapor-phase AITC has been distinguished for its excellent antifungal capacity in fruits at low concentration (3). It is known that the volatile compounds coming from essential oils are the main reason for their antimicrobial properties (4). The advantage of these active volatile compounds is that they can penetrate in most of the food matrixes, favoring their action from the headspace to the food surface. For this reason, AITC has been considered an excellent option to the design of active food packaging, which requires a microbiological control over the entire food surface. Indeed, Bahmid et al. demonstrated that the release of this compound from a cellulose acetate film was directly related with the sorption capacity of AITC in different types of beef, producing different antimicrobial effects on them (5). Likewise, active materials containing AITC have been validated in many different food stuffs, such as meat products (6), bakery (7, 8), fruits, and vegetables (9). However, it has also been reported that high volatility of AITC could prevent a lasting effect over time (10).

To improve the antimicrobial efficacy of volatile compounds, the complexation with cyclodextrins has been widely applied and reported (11–14). Cyclodextrins are cyclic oligosaccharides with a toroidal conic shape composed by 6, 7, or 8 (alpha, beta, or gamma-cyclodextrin) glucose units linked by α-1,4 glycosidic bonds (15). The external wall of these molecules is hydrophilic, as a result of primary and secondary hydroxyl group positions that favor its solubility in water. In contrast, the glycoside-bonds are oriented toward the internal cavity, which made it more hydrophobic. Due to this, cyclodextrins are able to form host:guest inclusion complexes (IC) with a wide variety of hydrophobic molecules (11, 16).

The encapsulation properties of an IC can lead to an increase of the water solubility and thermostability of a guest compound or to a masking effect of its odor or flavor due to the interaction with the cyclodextrin cavity (16). Moreover, one of the most attractive properties about IC is related to the control on the release of the guest compounds (11). This fact can be the key to the design of active packaging materials, where it is required to maintain a specific activity over time. In this regard, the versatility of the IC could allow their incorporation in packaging materials, such as polymer films, fibers, nanofibers, hydrogels, and aerogels (17–19). Therefore, they could be derived in several active packaging systems, such as bags, trays, sachets, pads, labels, among others (20).

Within the cyclodextrins family, beta-cyclodextrin (β-CD) has been the most studied and used to the formation of IC due to its low cost, good availability, and the cavity size (6.0–6.5 Å diameter) that allows the complexation of a wide range of hydrophobic compounds (16). Furthermore, one of the aspects that differentiate notably the β-CD from the other cyclodextrins is the low water solubility (21). This difference in the water solubility is due to the existence of high intramolecular interactions and also because β-CD tends to retain water molecules around it and inside the cavity (22). Thus, the relative humidity (RH) would influence significantly the guest compound release as was evidenced in a previous study of β-CD with eugenol and cinnamaldehyde (12).

**Figure 1:**
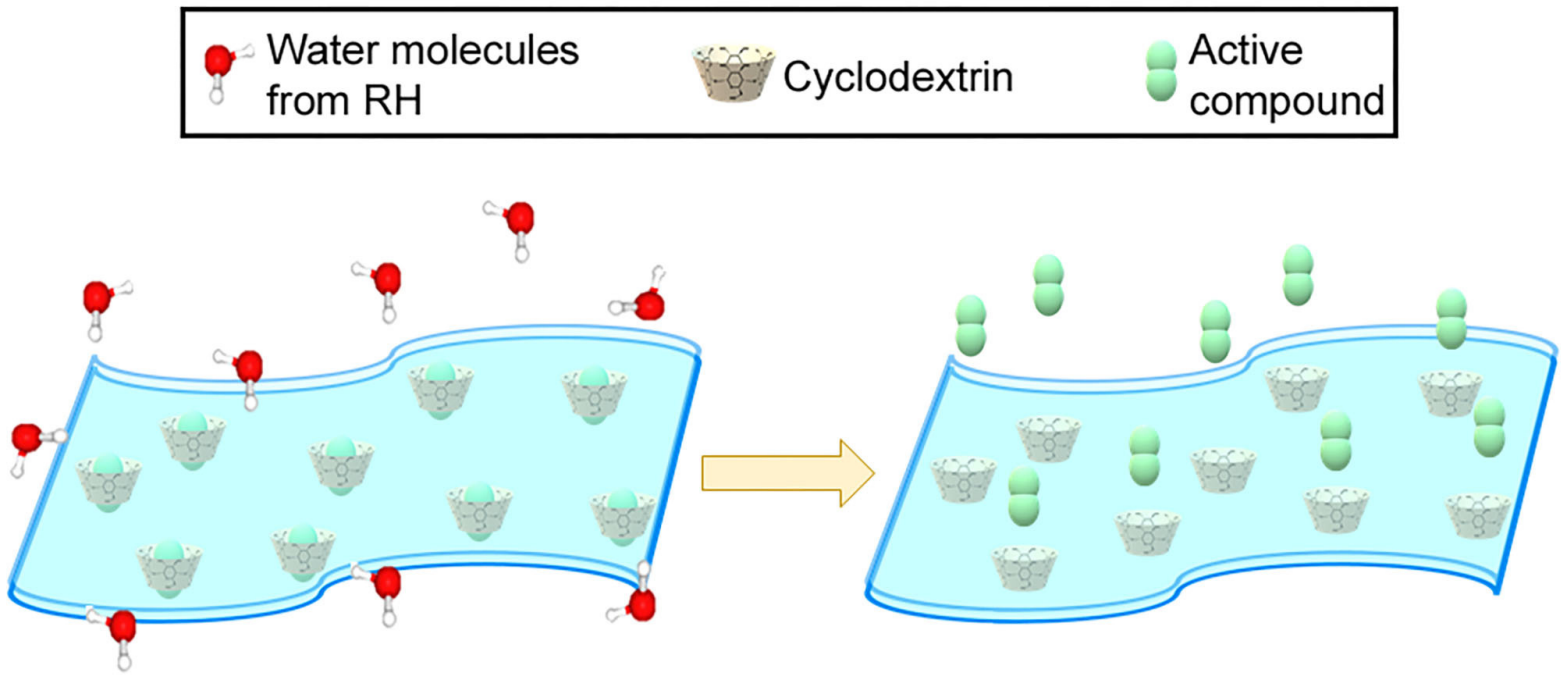
RH-responsive capacity of the IC inside a packaging material.

On the other hand, RH is also a very important extrinsic factor that determines the quality of food (23). In this way, the IC with β-CD could be useful for the design of RH-responsive antifungal food packaging. Unlike common active food packaging materials, responsive food packaging materials are activated as response to changes in the food or the surrounding atmosphere conditions, such as RH (24). Thus, the incorporation of β-CD IC in an RH-responsive packaging material could maintain its functionality at low RH, while high RH could trigger compound release (Figure 1). In consequence, an active atmosphere could be generated in order to avoid food decay during storage and distribution, which is one of the actual challengers in active food packaging engineering (25).

Based on the above, the aim of this work was to study the AITC release from β-CD:AITC IC to headspace during the exposure from different percentages of RH and to evaluate how this release favors the generation of an active atmosphere that is able to control *Botrytis cinerea* growth.

## Materials and Methods

### Materials

β-CD pharma grade (MW = 1,134.98 g mol^−1^, 98.5% purity) was obtained from Cyclolab, Ltd. (Budapest, Hungary). AITC (MW = 99.15 g mol^−1^) with 95% purity, and butylated hydroxytoluene (BHT) as a stabilizer (<0.1%) was purchased in Sigma Aldrich (Santiago, Chile). Acetonitrile with HPLC grade and technical grade solvents were acquired from Merck S.A. (Santiago, Chile). *Botrytis cinerea* (B05.10 strain) was gently provided by Center of Plant Biotechnology, Faculty of Biological Science, Universidad Andres Bello, Chile.

### IC Preparation

Two different β-CD:AITC initial molar ratios were considered (2:1 and 1:1) in order to evaluate the differences in entrapment efficiency of IC. The IC was prepared by co-precipitation according to a previously reported method with some modifications (12). Briefly, 5 g of pure β-CD were put in an amber flask with 50 mL of ethanol: water 1:2 (v/v) and stirred at 50°C for 2.5 h. Then, the corresponding amount of pure AITC (according to the β-CD:AITC molar ratio) was added to the flask, and it was hermetically sealed to avoid the volatilization of the compound. The mixture was stirred with a magnetic stirrer for 30 min at 50°C; then, it was cooled at room temperature for 1 h and refrigerated overnight. The solid phase was recovered by vacuum filtration and washed with 10% (v/v) ethanol solution to remove the possible superficial AITC. This recovered solid phase was dried in a CoolSafe 55-4 Pro freeze dryer (Labogene, Denmark). Finally, the fraction below 45 μm was selected using a #325 Tyler sieve. Samples were kept in hermetic glass flasks and stored in desiccators containing activated silica gel at 25°C until further characterization.

### Physical Mixtures Preparation

PM with molar ratios β-CD:AITC of 1:1 and 2:1 were prepared. Freeze dried β-CD and pure AITC were mixed with a spatula in a ceramic mortar until a homogeneous mixture was obtained.

### Verification of the IC Formation

#### Entrapment Efficiency and AITC Content in IC

To determinate complexed AITC content, the active compound was extracted from 5 mg of IC with acetonitrile (5 mL) in hermetically sealed vials, using an IST-4075 incubator shaker (Jeio Tech, Korea) at 150 rpm and 25°C for 24 h. The insoluble phase (β-CD) was separated by centrifugation, and the supernatant was analyzed using a Perkin Elmer Clarus 500 gas chromatographer system with a flame ionization detector (FID) and a ZB-50 capillary column (30 m × 0.53 mm ID, 1.00-μm film thickness, Phenomenex, Torrance, USA). The volume injection was 1 μL in a 1/2 split mode. The oven temperature started at 60°C for 1 min, and then raised to 200°C at 20°C min^−1^; after 2 min, the temperature is increased at 20°C min^−1^ until 240°C, then kept for 0.5 min. Helium was used as carrier gas at 50 mL min^−1^. The injection and detector temperature were fixed at 80 and 290°C, respectively. The compound was quantified using a calibration curve previously prepared from eight solutions of AITC in acetonitrile with concentrations between 0 and 100 ppm (*R*^2^ = 0.99). From this analysis, it was possible to determine the EE according to Equation 1 (13).


(1) $$\begin{array}{l} EE\;\left(\%\right)=\;\frac{released\;AITC\;content}{theoretical\;AITC\;content}\;\times \;100 \end{array}$$

where *real complexed AITC content* was the AITC content in the IC, and *theoretical AITC content* corresponded to the initial AITC amount used to prepare the IC. The experiments were carried out in triplicate, and the average was reported.

#### Thermogravimetric Analysis

The TGA of β-CD, AITC, IC, and PM were carried out on a TGA/DSC 1 analyzer (Mettler Toledo, Switzerland). Samples (≈6 mg) were heated in an alumina crucible (70 μL) from 30 to 700°C at a rate of 10°C min^−1^ in a nitrogen atmosphere. TGA was performed in duplicate.

#### X-Ray Diffraction

The XRD patterns of freeze-dried β-CD and IC were obtained with a Diffractometer D5000 (Siemens, Germany) at 30 mA and 40 kV with CuKα (*k* = 1.54 Å) radiation at room temperature and 0.02° s^−1^ step size in a 2θ range of 2–20°. It is important to note that the XRD pattern of AITC was not acquired since it is a liquid substance, and, therefore, it produces no diffraction patterns (26).

#### Fourier-Transform Infrared Spectroscopy

FTIR spectra of β-CD, AITC, IC, and PM were obtained using an Alpha IFS 66 V spectrometer (Bruker, Germany). The spectra were acquired from 4,000 to 400 cm^−1^ with 64 scans by the KBr disc technique.

#### Proton Nuclear Magnetic Resonance (^1^H-NMR) Spectroscopy

Samples of β-CD and powder IC (10 mg) were dissolved in DMSO-d6 (0.7 mL) that contained tetramethylsilane (TMS) as reference (27, 28). Then, 200 μL of the solution were transferred to 5-mm ^1^H-NMR tubes, and spectra were acquired using an Ultrashield NMR spectrometer (Bruker, Switzerland) at 300 mHz and 300 K.

### IC Characterization

#### Water Sorption Capacity

The water sorption capacity of freeze-dried β-CD, and IC was determined using the standard isopiestic static-gravimetric method (29). For this, 125 mg of each sample was put separately in 30-mm diameter plates at 20°C and 23, 50, or 97% RH. Desiccators with saturated salt solutions of potassium acetate, magnesium nitrate, and potassium sulfate were used to maintain constant RH. The samples were weighted in an electronic analytical balance one time a day until constant weight (±0.001 g), condition that was reached after 7 days. The change in water content was determined in a dry basis. The experiments were carried out in triplicate; average was reported.

#### Antifungal Activity Against *Botrytis cinerea*

As the AITC is a volatile compound, a vapor-phase experiment was designed to evaluate the antifungal activity of IC against *B. cinerea*. For this, a 50-mm diameter potato dextrose solid agar (PDA) disc was put in the center of a sterile petri dish (150-mm diameter). Then, 10 μL of *B. cinerea* spore suspension (10^5^ spores/mL) was inoculated in the middle of PDA disc. Subsequently, 20 mg of β-CD (control of β-CD) and different amounts (4, 6, 10, and 20 mg) of IC distributed in 2 plates (30-mm diameter) were put around the agar disc without contact (see Supplementary Figure 1). The different amounts of IC and the corresponding concentration of AITC calculated based on 0.2 L of air inside the plate (headspace) are detailed in Table 1. A petri dish without IC was used as a control sample. Finally, the system was sealed with Petrifilm^®^ to avoid AITC leakage and stored at 20°C. The radial fungal growth was measured until the fungus covered the entire surface of PDA disc in the control sample (5th day). The radial inhibition (% RI) was calculated according to Equation 2.


(2) $$\begin{array}{l} \text{RI}\left(\%\right)=\left(X_{C}-X_{CI}\right)/X_{C}\;*\;100 \end{array}$$

where *X*_*c*_ was the fungal-growing halo (mm) of the control sample, and *X*_*CI*_ was the fungal-growing halo (mm) of the petri dish with β-CD or IC. The antifungal assay was carried out in triplicate, and average was reported.

**Table 1:** Antifungal assay conditions of β-CD:AITC IC against *B. cinerea*.

**IC amount (mg)**	AITC concentration inside the plate^**^ (ppm)	
	**β-CD:AITC_2:1**	**β-CD:AITC_1:1**
0^*^	–	–
4	0.66	1.03
6	0.99	1.55
10	1.64	2.59
20	3.29	5.17

* *20 mg of β-CD (control of β-CD)*.

** *Calculated based on real AITC content in IC (section Verification of the IC Formation) and 0.2 L of air as the headspace in the plate*.

### AITC Release From IC

The same saturated salt-water solutions (0.6 mL) used for water sorption capacity determination were prepared into 22-mL headspace vials and conditioned at 20°C for 30 min. Then, a sample of 0.2 mg of IC was put into a 0.3-mL flask, and it was put inside headspace vials (see Supplementary Figure 2). Headspace vials were sealed (silicone/PTFE septum and an aluminum hole cap) and stored at 20°C. To determine the AITC headspace concentration, the vials were analyzed at different times using a Perkin Elmer Turbomatrix 40 headspace analyzer, which was coupled to a Clarus 580 gas chromatographer equipped with the ZB-50 capillary column described in section Entrapment Efficiency (EE) and AITC Content in IC. The vials were placed on the headspace autosampler system and thermostated for 10 min. Needle and transfer line temperature in headspace was set at 35 and 45°C, respectively. Headspace samples of 0.2 mL were transferred to gas chromatographer where the oven started at 40°C for 0.5 min; it increased at 20°C min^−1^ until 120°C; after 0.5 min, the temperature raised at 20°C min^−1^ to 270°C, and it remained for 0.5 min. Helium was used as carrier gas at 54.5 mL min^−1^. The injection and FID detector temperature were fixed at 240 and 260°C, respectively. The released AITC was quantified using a calibration curve prepared by the procedure described in section Calibration Curve for AITC Release Determination. The % of released AITC in each time was calculated using Equation 3.


(3) $$\begin{array}{l} Released\;AITC\;\left(\%\right)=\;\frac{released\;AITC\;}{real\;complexed\;AITC\;content}\;\times \;100 \end{array}$$

where *released AITC* was the released AITC from the IC, and *real complexed AITC content* was the AITC content in 0.2 mg of IC, determined in section Entrapment Efficiency (EE) and AITC Content in IC. The experiments were carried out in triplicate, and average was reported.

#### Calibration Curve for AITC Release Determination

Eight solutions with different concentrations of AITC in acetonitrile were prepared. Then, 5 μL of each solution was injected through the septum of sealed headspace vials (22 mL). Thus, concentrations from 0 to 0.5 ppm in the headspace were achieved. The vials were analyzed through the headspace-gas chromatography method described in section AITC Release From IC. Each point was carried out in triplicate. The average of the area behind AITC peak in the chromatogram vs. AITC concentrations in the headspace was plotted to obtain the calibration curve (*R*^2^ = 0.97).

#### Molecular Dynamic Studies

The molecule dockings and IC conformation were acquired and selected with the procedure described in a previous study (12). The MD simulations of two conformations were performed using NAMD 2.6 (30) with the Charmm33b force field (31). The molecules were previously parametrized by ACPYPE web portal application at http://webapps.ccpn.ac.uk/acpype (32), and a water box of 50 x 50 x 50 Å with 3,837 water molecules was employed to soak each IC conformation. A general protocol was used with a cutoff value of 14 Å, followed by 100,000 steps of minimization and 50 ps of heating (0–298 K), comprising an initial phase of equilibrium and, finally, 140 ns of recording data of the molecular dynamics. Data were analyzed using the Qtiplot software.

### Statistical Analysis

Statistical analysis was carried out by using InfoStat software Student version (Universidad de Córdoba, Argentina). Paired Student *t*-test was applied to determine differences in the entrapment efficiency and in an antifungal assay. In addition, water sorption capacity of β-CD, β-CD:AITC_2:1 and β-CD:AITC_1:1 was compared through analysis of variance (one-way ANOVA), followed by Tukey test. All analyses were considered significant differences at the level of *p* < 0.05.

## Results and Discussion

### Verification of the IC Formation

#### EE and AITC Content in IC

Table 2 resumes the AITC content in the different developed IC and the associated EE. These results evidenced that the initial molar ratio had a significant effect in such parameters. A higher EE % was evidenced when the 2:1 molar ratio was used compared to 1:1. This may be because the excess of β-CD molecules could offer a higher probability to complex AITC molecules. Similar results were founded by Ohta et al. (33), where lower rate constants of AITC decomposition were reached when higher concentrations of β-CD were added. Moreover, this fact could also be associated with the stoichiometry of formed IC. According to Zhang et al. (34), a 1:1 stoichiometry is suitable for β-CD:AITC IC due to the structure of the guest compound. In this regard, EE near to 100% was expected for both β-CD:AITC_2:1 and β-CD:AITC_1:1. However, this EE % was not achieved probably due to decomposition of some AITC by its exposition to aqueous media (33). Moreover, it is important to consider the volatilization of AITC at 50°C, reducing the AITC concentration that is available in the aqueous medium, and as a result, generating a lower EE in the IC. Despite this, the EE values were higher than those reported in a previous research for compounds with a linear structure similar to AITC (13). In comparison with this last study, the increase from 30 to 50°C during active compound addition could favor the AITC complexation due to higher solubility of β-CD at higher temperature (35). Furthermore, the lower polarity and higher size of the hydrophobic allyl part of AITC would facilitate the interaction with the cavity of the β-CD. By contrast, AITC showed a lower EE than other compounds with a high volume section, such as aromatic rings, cycloalkane, or cycloalkene structures (36, 37). In a previous article, it was shown that the volume of the hydrophobic group of the guest molecule is essential to favor the intermolecular interactions with cyclodextrin cavity (12), which was confirmed by this study. However, as was discussed above, better performance (high EE) in AITC complexation with β-CD also depended on another factor such as the initial molar ratio and preparation conditions.

**Table 2:** AITC content and EE of β-CD:AITC IC.

**Molar ratio β-CD:AITC**	**AITC content (mg_**AITC**_/g_**IC**_)**	**EE (%)**
2:1	32.93 ± 0.85^b^	78.83 ± 2.38^a^
1:1	49.63 ± 1.61^a^	61.53 ± 2.05^b^

*Main values with the same superscript letter (a or b) in each column indicate no statistically significant differences in the parameter (p > 0.05) according to Student's T-test*.

#### TGA

**Figure 2:**
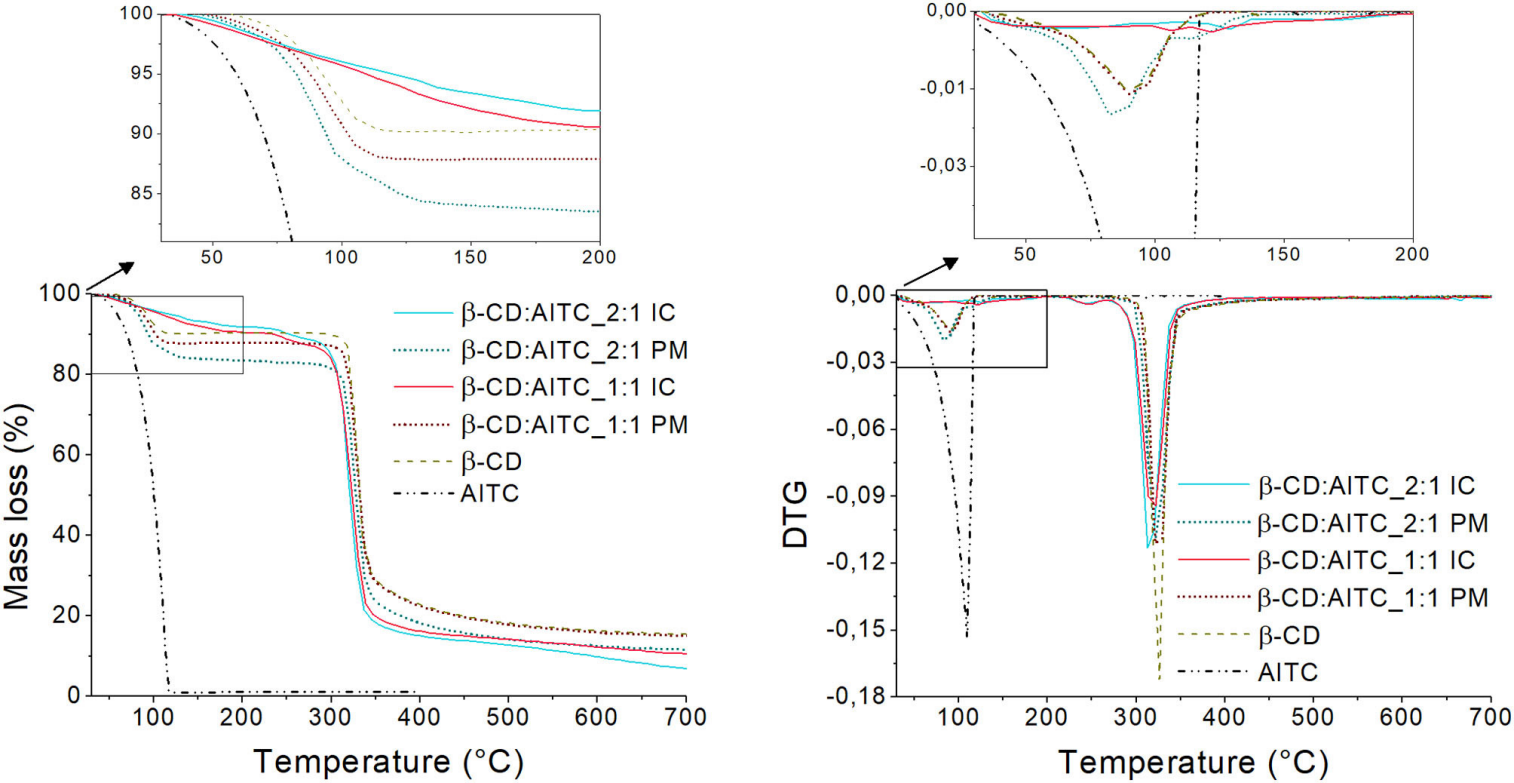
TGA thermogram and the DTG curve of AITC, β-CD, β-CD:AITC PM, and IC.

According to Figure 2, AITC evaporation was centered at 105°C during TGA analysis. Moreover, thermogram of β-CD showed two phases: (i) dehydration at 82°C, and (ii) thermal degradation of the cyclodextrin ring at 325°C, which is in accordance with other authors (38, 39).

On the other hand, the thermograms of IC were compared to PM to verify the effective AITC thermal protection by β-CD and also to analyze the thermal stability of IC. In all IC and PM thermograms and DTG, the maximum mass loss was centered at around 320°C, which corresponds to the β-CD thermal decomposition. Moreover, PM showed a thermal phase between 90 and 100°C related to the evaporation of AITC (zoomed zone). However, this phase did not appear in IC, which confirmed the AITC complexation. Conversely, the IC presented a good thermal stability (higher than 300°C). This fact allows to ensure the use of IC in high temperature processes, such as the extrusion for the development of polymeric packaging materials.

#### XRD Analysis

**Figure 3:**
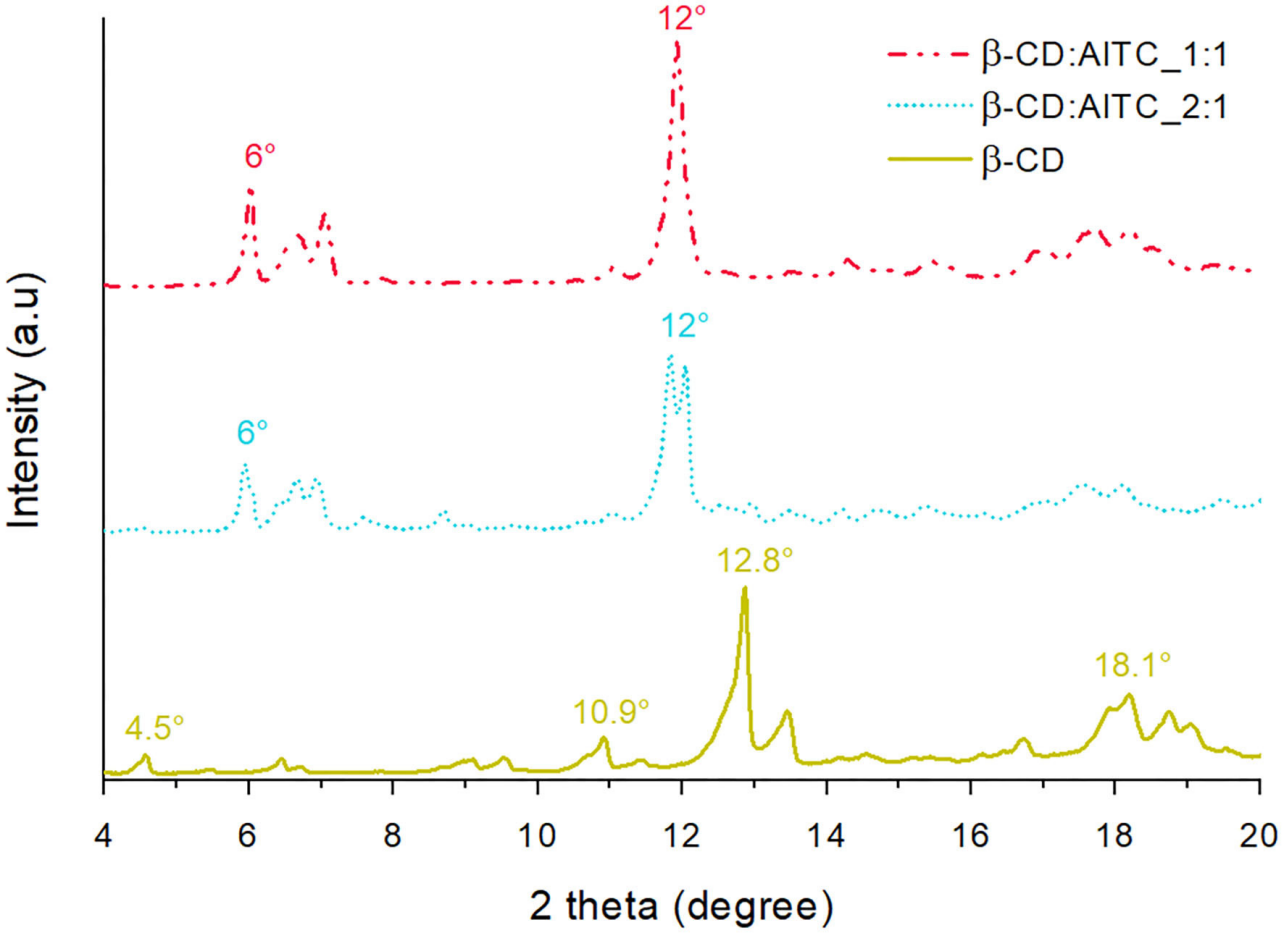
XRD patterns of freeze-dried β-CD and β-CD:AITC IC.

Figure 3 shows the XRD diffractogram of freeze-dried β-CD with the characteristic bands at 4.5, 10.9, 12.8, and 18.1° in the 2θ value (27, 28). In the case of IC, the peaks at 4.5 and 18.1° disappeared, new peaks between 6 and 7° were registered, and the signal at 12.8° in the β-CD pattern shifted to 12° in IC. In general, these changes in the intensity and position of peaks could be related with the host:guest interaction that is interpreted as evidence of AITC complexation with β-CD (40). Furthermore, the diffractograms evidenced a change in the packing arrangement of the molecules. According to the requirements described by Tanwar et al. (22), the pure β-CD showed an XRD pattern of an “herringbone” packing type, while the β-CD:AITC samples exhibited a “channel” packing arrangement, being this the typical structure of an IC with β-CD (15).

#### FTIR Spectroscopy

**Figure 4:**
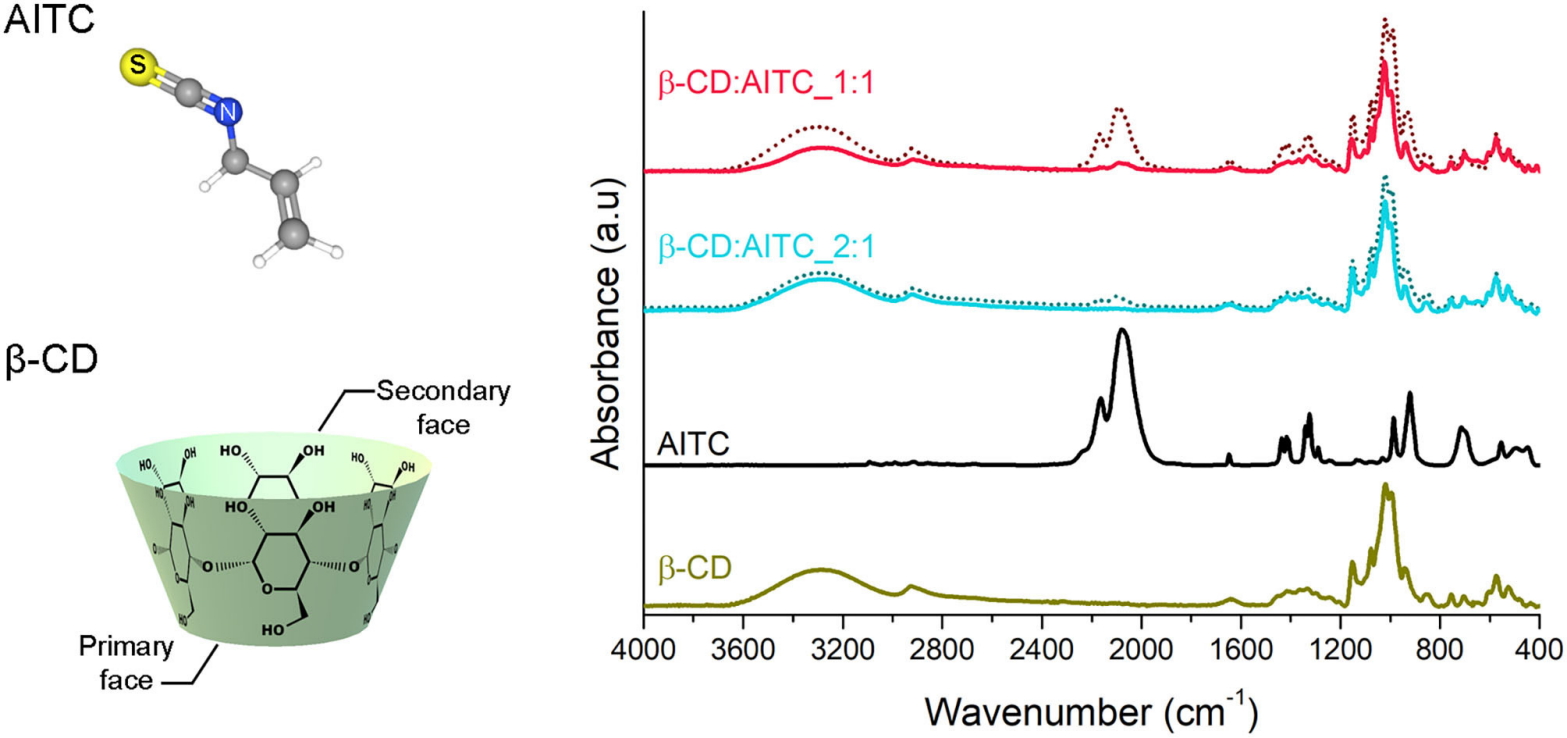
Chemical structure of AITC, β-CD, and FTIR spectra where the solid line corresponds to IC and the pointed line to PM.

In order to obtain information about the AITC complexation with β-CD, the IC and PM were analyzed by FTIR spectroscopy (Figure 4). The pure β-CD spectrum showed bands at 3,274, 2,925, 1,644, 1,152, 1,077, and 1,020 cm^−1^ related to hydroxyl groups (–OH stretching), methylene groups (C–H stretching), H–O–H bending of hydroxyl groups, glycoside skeleton (–C–O– stretching), and the secondary and primary O–H bending of hydroxyl groups, respectively (41, 42). On the other hand, the AITC spectrum showed bands at 2,080, 1,416, and 920 cm^−1^ that were assigned to isothiocyanate group stretching (–NCS), –CH-CH– shear, and R–C-CH_2_ bending of the allyl group, respectively (43, 44). The spectra of PM showed the characteristic bands of pure β-CD and AITC, evidencing no interaction between them. Conversely, the spectra of IC revealed mainly absorption bands associated with the β-CD structure; however, it was possible to observe small displacement in some of them. Thus, the bands corresponding to glycoside were slightly displaced to 1,155 cm^−1^. This behavior could be explained due to the interaction between the hydrophobic part of AITC and the internal cavity of the β-CD. In addition, small bands associated with the isothiocyanate group of AITC at 2,080 cm^−1^ were displaced to 2,090 cm^−1^ for β-CD:AITC_1:1 IC, and secondary and primary –OH bonds of β-CD were slightly displaced to 1,079 and 1,023 cm^−1^, respectively. These observations could demonstrate a possible interaction between the –NCS group located outside the β-CD cavity and the external –OH group corresponding to the cyclic oligosaccharide. In this regard, the -NCS group could be oriented on either side on β-CD (primary or secondary face), leading to different IC structures (see Supplementary Figure 3). Furthermore, these proposed structures are consistent with those described by Ohta et al. for IC of AITC with α-CD (45).

#### ^1^H-NMR Spectroscopy

The ^1^H-NMR spectroscopy is a technique that allows to know β-CD:AITC IC structures (44). Therefore, this technique was used in order to stablish possible IC structures and to relate them with the AITC release process. The obtained ^1^H-NMR spectra and labeling of β-CD protons were added as Supplementary Figure 4. The corresponding chemical shifts (δ) of the protons in β-CD and β-CD:AITC IC are resumed in Table 3. Since δ values were equal for β-CD:AITC_2:1 and β-CD:AITC_1:1, showing that the molecular structure was the same independently of the initial molar ratio, only one Δδ value was presented in Table 3. The δ values of H-1, H-2, H-4, and H-6 in IC were similar to those of β-CD. In contrast, 10-fold higher Δδ values were observed for H-3 and H-5 with 0.0018 and 0.0029 ppm, respectively. These changes would indicate a modification of the β-CD cavity. According to literature, these differences in Δδ values between IC and β-CD are the best evidence to verify the formation of a cyclodextrin IC (46). Moreover, these values correspond to electronic shielding, which indicates that there were atoms that interacted with the mentioned protons. According to FTIR spectroscopy observations, these atoms could correspond to the allyl group of AITC molecules. Moreover, OH-3 showed a high downfield shift. This could indicate that more electronegative atoms were surrounding this outside group of β-CD (47). Once again, these findings are consistent with FTIR spectroscopy observations, where the –NCS group of AITC seemed to be exposed to the β-CD secondary face. Similar observations have been reported for IC of AITC with α-CD, where the polar region of the guest compound was preferably oriented to the wider ring of the cyclodextrin (48).

**Table 3:** ^1^H-NMR chemical shift values for β-CD and β-CD:AITC IC in DMSO-d_6_ at 300 mHz and 300 K.

Proton^*^	**Chemical shift**		
	**β-CD (δ_0_)**	**β-CD:AITC (δ)**	**Δδ (δ – δ_0_)**
H-1	4.8314	4.8311	−0.0003
H-2	3.3005	3.3009	0.0004
H-3	3.6321	3.6339	0.0018
H-4	3.3497	3.3502	0.0005
H-6	3.6608	3.6602	−0.0005
H-5	3.5747	3.5776	0.0029
OH-2	5.7190	5.7189	−0.0001
OH-3	5.6651	5.6632	−0.0019
OH-6	4.4353	4.4355	0.0002

* *Protons are labeled in the Supplementary Figure 4*.

### IC Characterization

#### Water Sorption Capacity

**Figure 5:**
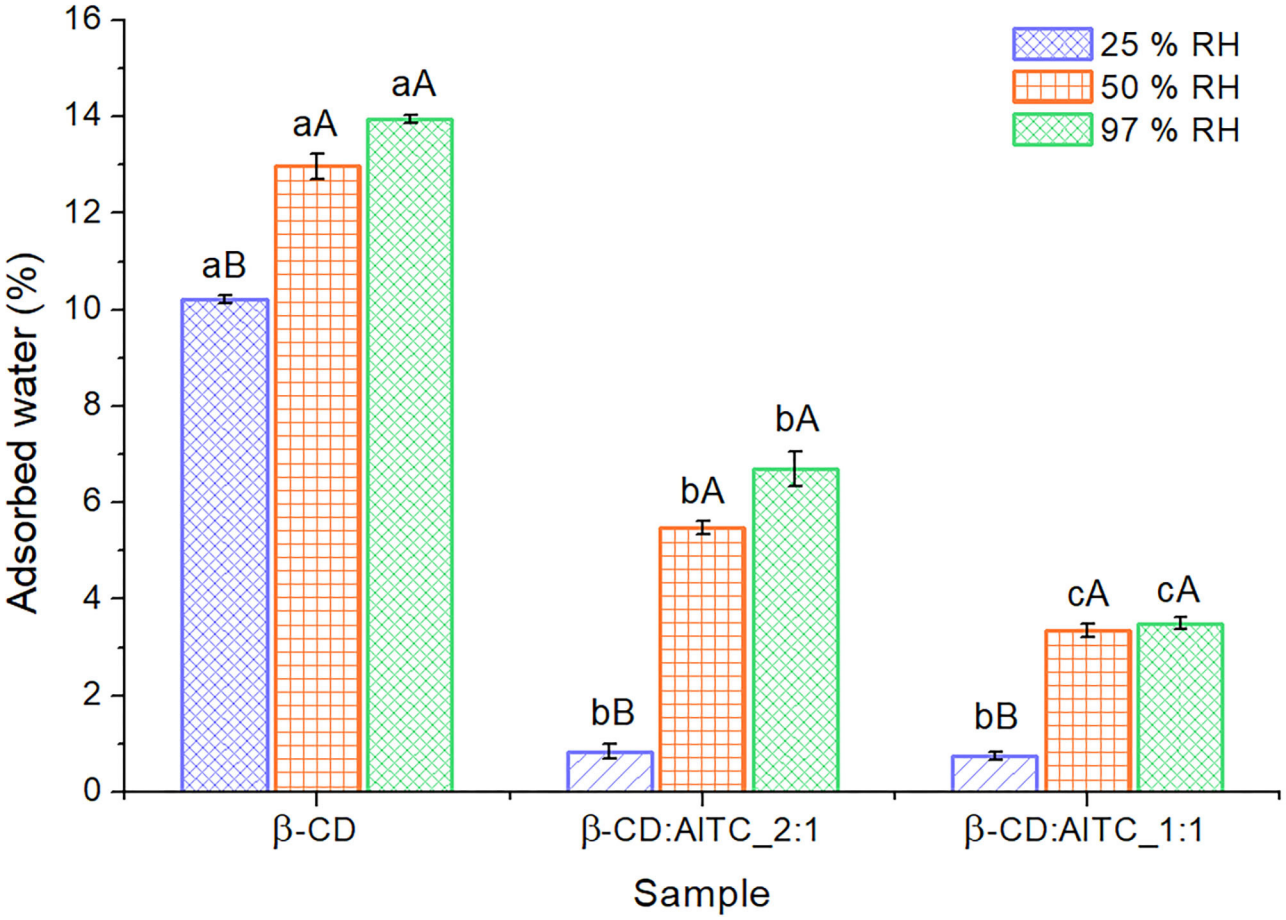
Water content of β-CD and β-CD:AITC IC after exposition at different percentages of RH and 20°C for 7 days. According to ANOVA and Tukey tests, different lowercase letters (a, b, and c) indicate statistically significant differences (*p* < 0.05) between samples at the same RH, while different capital letters (A and B) show statistically significant differences (*p* < 0.05) in the sample by the RH change.

Figure 5 represents the average of water content in samples after 7-day exposure at different percentages of RH at 20°C. It was found to have a higher water sorption capacity in all samples from 50% RH (capital letters). Moreover, pure freeze-dried β-CD resulted in a higher water content than IC at all RH (lowercase letters). This fact can be explained because β-CD is able to accommodate water molecules both inside and outside of it due to the presence of different primary and secondary hydroxyl groups in the glucopyranose structure (49, 50). However, when the IC was formed, the availability of the hydroxyl groups oriented to the inner cavity of the β-CD was reduced by the presence of AITC. In addition, as is shown in the Supplementary Figure 5, the surface of β-CD was changed by the formation of IC. Here, IC showed a less porous surface, which could possibly affect the water sorption capacity of the β-CD.

On the other hand, β-CD:AITC_2:1 presented a higher water content than β-CD:AITC_1:1 (Figure 5). However, this effect could be also related with the assumption of the stoichiometry 1:1 in the IC, since the β-CD:AITC_2:1 sample could contain β-CD molecules without AITC; as a result, they would be able to sorb more water molecules.

#### Antifungal Capacity

**Figure 6:**
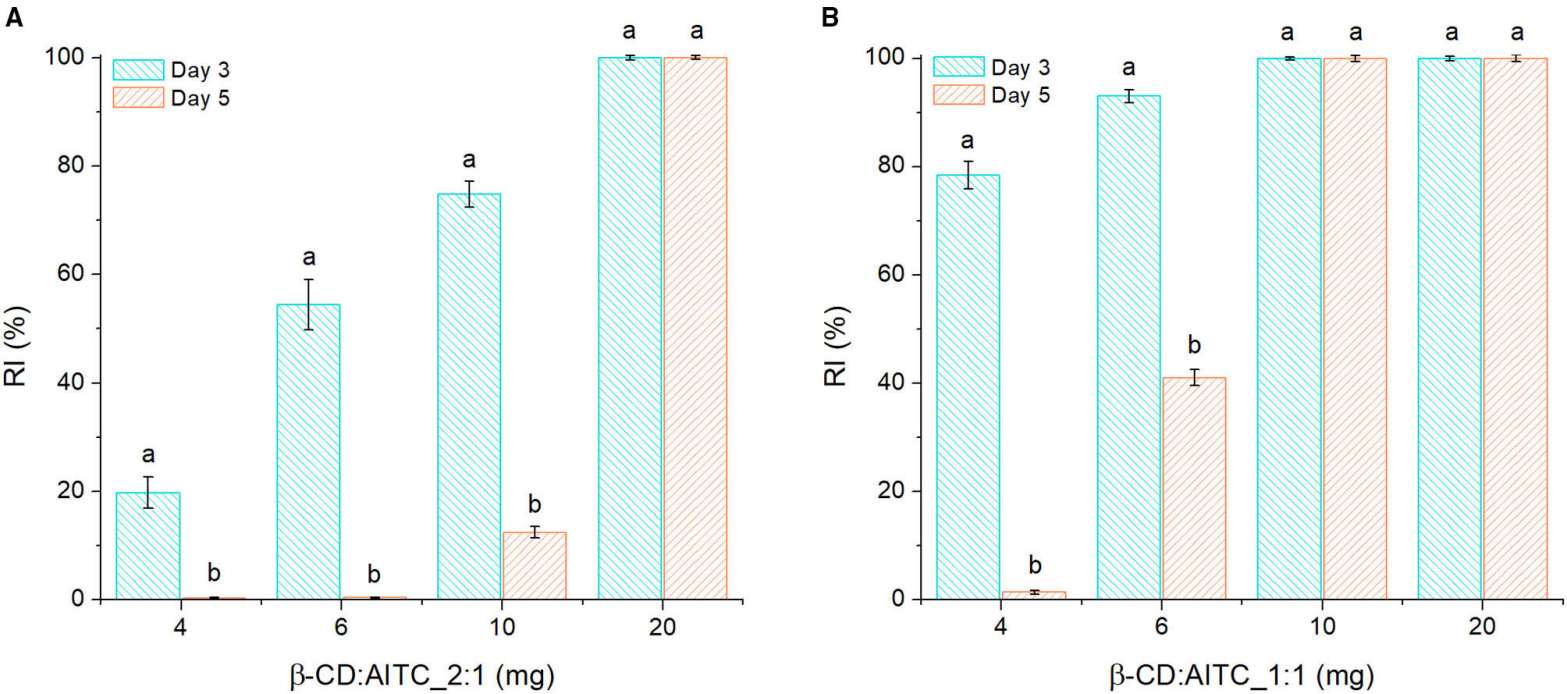
Inhibition of *B. cinerea* growth by different amounts of **(A)** β-CD:AITC_2:1 and **(B)** β-CD:AITC_1:1 in a headspace system. Different letters (a and b) show statistically significant differences (*p* > 0.05) between Days 3 and 5 for the same evaluated sample amount.

Figure 6 summarizes the antifungal activity (expressed as RI %) of β-CD:AITC_2:1 (Figure 6A) and β-CD:AITC_1:1 (Figure 6B) against *B. cinerea* after 3 and 5 days of exposure in a headspace system at 20°C. Normal growing of *B. cinerea* was evidenced with β-CD (% RI = 0.1 ± 0.02); by contrary, IC affected the fungal growth according to the exposed amount. Thus, although 4, 6, and 10 mg of β-CD:AITC_2:1 or 4 and 6 mg of β-CD:AITC_1:1 inhibited *B. cinerea* growth until 3rd day, the effectiveness in these samples was significantly reduced at Day 5 (*p* > 0.05). On the contrary, when 20 mg of β-CD:AITC_2:1 or 10 mg of β-CD:AITC_1:1 were used, a total inhibition (100% RI) of fungal growth was reached and maintained over time. The difference in % RI with 10 mg of IC 1:1 and 2:1 ratio could be related with the AITC content shown in Table 1. In addition, IC from the last-mentioned samples was removed at Day 5, and no fungal growth was evidenced at Day 10 of the assay (see Supplementary Figure 6). These results evidenced the fungicide function of inclusion complexes against *B. cinerea*.

On the other hand, according to Table 1, the AITC headspace concentration was 2–3 ppm in samples where *B. cinerea* was totally inhibited. Ugolini et al. reported the use of similar concentrations of pure AITC in the vapor phase to prevent this fungus growth in fruits (3). Thus, it further reinforces that IC formation maintains the antifungal capacity of AITC at low concentrations and that its action is prolonged in time. Consequently, this IC could be considered as potential antifungal agents for the design of antifungal materials.

### AITC Release From IC

**Figure 7:**
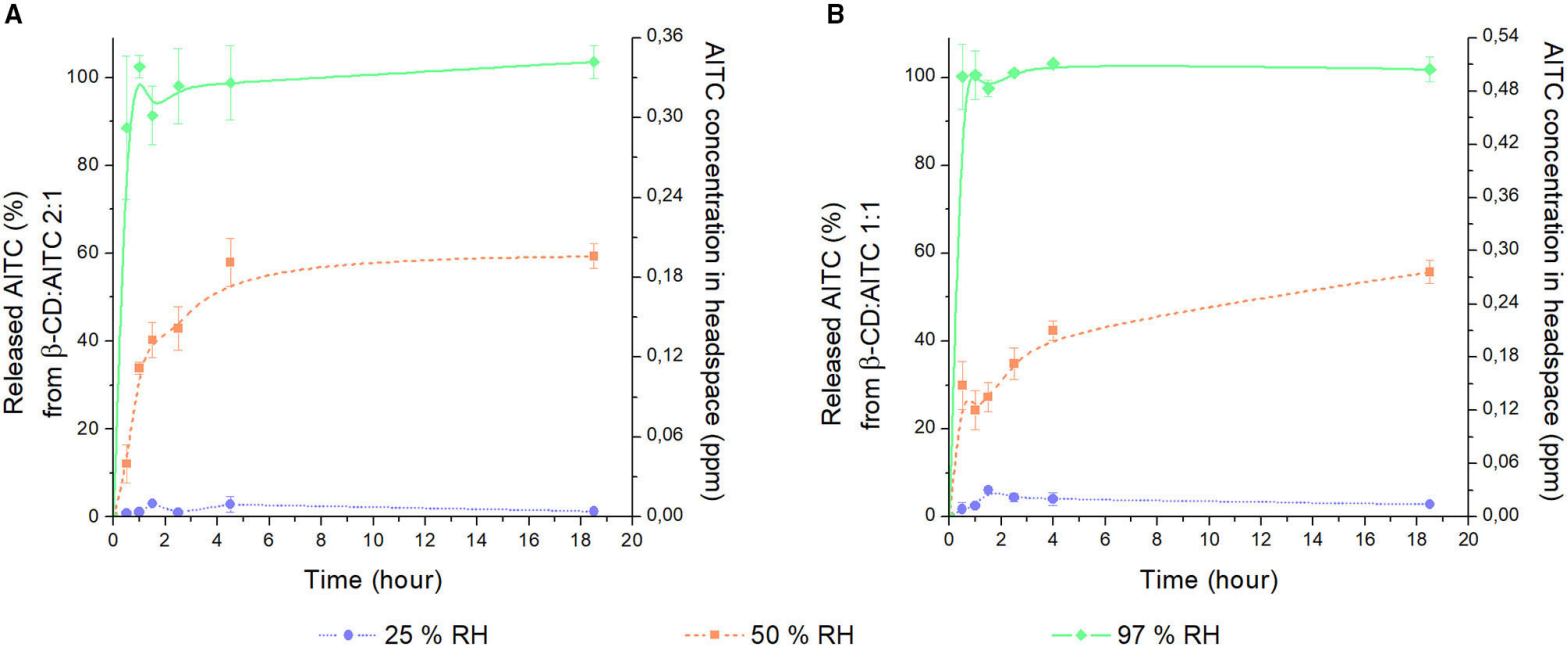
Percentage and concentration of released AITC from **(A)** β-CD:AITC_2:1 and **(B)** β-CD:AITC_1:1 IC at different percentages of RH and 20°C in a headspace system. Values are mean of three replicates, with error bars depicting the variation (standard deviation).

In order to explain the influence of RH on the AITC release from IC, it was exposed to different percentages of RH at 20°C. Figure 7 resumes the releasing curves of AITC during for 18 h. A clear effect of RH on the release of AITC from both IC was evidenced, and this effect was consistent with the water sorption of IC. To understand the mechanism of AITC release from IC, water sorption isotherms were graphed (Supplementary Figure 7). Herein, a plateau from 0.5 aw was observed for both β-CD:AITC_2:1 and β-CD:AITC_1:1, which evidenced the formation of a crystalline hydrate structure (51). This hydrate was probably achieved by the “sequential binding of the individual water” hydration mode reported by Pereva et al. (50). In this mode, the formation of a β-CD hydrate starts through the interaction between one water molecule (*n* = 1) and hydroxyl groups of the primary face of oligosaccharide. Then, subsequent water molecules interact with the already ligand water molecules and with the other hydroxyl groups of the narrow face of β-CD. This interaction produces the increase of the number of water molecules (*n* ≥ 2), generating the occlusion of the β-CD primary face due to hydrogen bond interactions. Therefore, RH over 50% could favor the release of the AITC from the IC due to movements produced in the β-CD structure by its hydration. Furthermore, the possible exposure of the AITC polar group outside the β-CD cavity (described in the FTIR spectroscopy results section) could increase the polarity of the media. This fact, in turn, could attenuate the enthalpy of IC formation, and, thus, a significant change in the conformational structure of the β-CD could be produced (50).

**Figure 8:**
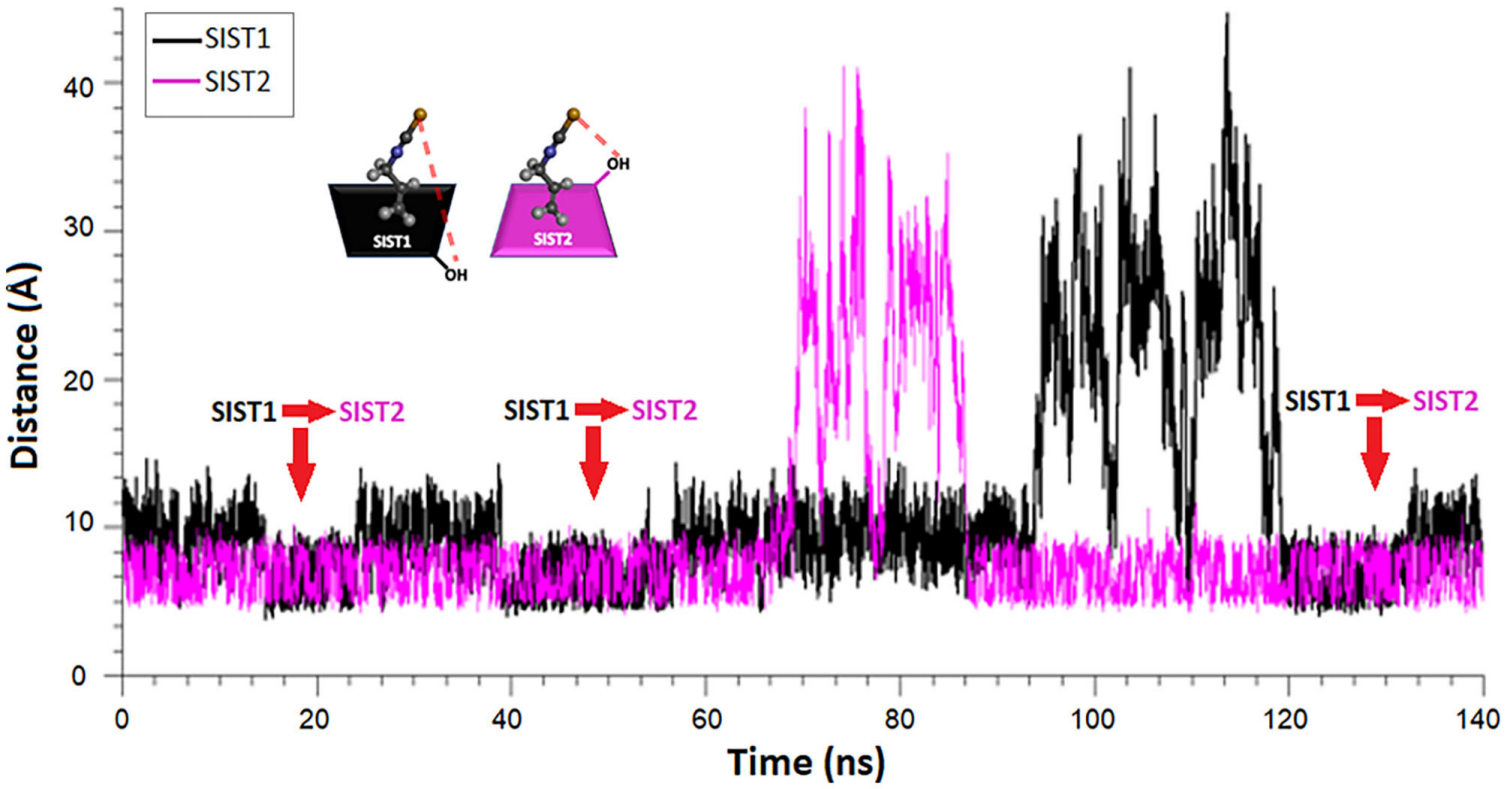
Relative position of AITC in the IC by MD.

On the other hand, Figure 7 shows that AITC release occurred in three stages: (i) a burst initial release, (ii) a slight drop of the release, and (iii) a slight final release until reaching the equilibrium. These stages could be associated with the behavior of water sorption of the SIST1 and SIST2 structures as represented in Supplementary Figure 3. For SIST2, the -NCS group of AITC oriented to the primary face of β-CD could favor the easy interaction between water molecules and the narrow face of the β-CD, prompting its significant distortion (50) that resulted in the fast release of AITC. Therefore, the first stage of AITC release could be associated with the IC with a SIST2 structure. Subsequently, the total release of AITC from this structure or a change in its conformation (explained in DM studies) could be related to the drop of compound release (Stage 2). Furthermore, another possible explanation for this drop is the presence of a higher amount of water in the system that could favor the complexation of AITC in the IC (52). After this stage, the IC with SIST1 structure where the -NCS group was oriented to the secondary face of β-CD could remain in the IC samples due to its better stability in the presence of water over time (see DM results, SIST1 in Figure 8). Thus, the last stage of AITC release was probably associated with SIST1 structure because the binding of individual water molecules could produce a slight “push” effect of AITC due to the occlusion of the primary face of β-CD. Furthermore, as SIST1 structure was more stable in water, a low rate of AITC release was obtained in this stage.

On the other hand, there was no difference between the total percentage of released AITC from β-CD:AITC_2:1 and β-CD:AITC_1:1, probably due to the presence of the same structures (SIST1 and SIST2) in both samples. In this regard, it is important to point that the β-CD:AITC_2:1 released 1, 59, and 100% of AITC content at 23, 50, and 97% RH, respectively; while β-CD:AITC_1:1 released 3, 56, and 100% of AITC at abovementioned RH. This is strong evidence to support that RH is a very important factor that is able to control the AITC release from β-CD:AITC IC to headspace. This behavior is useful for the design of antifungal food packaging with responsive capacity to RH, and to recommend the better conditions to maintain the activity of packaging material before its use.

#### MD Studies

MD studies were carried out in order to understand the AITC release from the different proposed IC structures during 140 ns. The MD results were analyzed; after that, complete stabilization of the two simulated systems was reached. Docking studies allowed the selection of conformation of AITC and β-CD. Moreover, the final IC structures (SIST1 and SIST2) were obtained by the energy binding and lowest –Δ*G* values. Figure 8 shows that, at the beginning, the AITC position of SIST1 presented a reversion to SIST2, showing the coexistence of the two IC structures. However, AITC did not present this behavior in the SIST2, showing higher stability. Then, the AITC molecule was removed at approximately 70 ns from SIST1 and at approximately 90 ns from SIST2. This fact was observed in Figure 8 by the deviation of original coordinates compared with the new position along the simulation. Finally, the AITC in the SIST2 returned to its original position; however, in the SIST1, it returned as SIST2, and it started to change the IC structure again. These observations are coherent with the proposed structures in the FTIR and ^1^H-NMR analyses presented in Supplementary Figure 3. Moreover, the events explain the first fast AITC release and the slight drop in the experiment of AITC release from IC.

## Conclusions

AITC was successfully complexed with β-CD, forming IC with a 1:1 stoichiometry and a channel packing arrangement. The DM studies allowed to confirm the coexistence of two IC structures, which was according to the position of AITC in the IC as proposed by analysis of FTIR and 1H-NMR spectroscopy. The IC at low amounts (20 mg of β-CD:AITC_2:1 or 10-mg β-CD:AITC_1:1) was able to inhibit *B. cinerea* growth over time and showed a fungicide function. On the other hand, the water sorption of IC was depended on RH. Here, the water sorption behavior was intimately related to AITC release, confirming that the RH value significantly influenced on the guest compound release. This allows to suggest β-CD:AITC IC as potential antifungal agents for the design of food packaging materials, whose activity would be able to adapt to RH changes.

## Data Availability Statement

The original contributions presented in the study are included in the article/Supplementary Material, further inquiries can be directed to the corresponding author/s.

## Author Contributions

CM-S: conceptualization, methodology, validation, investigation, formal analysis, data curation, writing the original draft, visualization, and funding acquisition. FR-M: conceptualization, resources, writing, review, editing, supervision, project administration, and funding acquisition. CM: conceptualization, software, writing, review, and editing. AH: methodology. JB: resources. AG: project administration and funding acquisition. MG: resources and funding acquisition. All authors contributed to the article and approved the submitted version.

## Funding

This research was funded by Agencia Nacional de Investigación y Desarrollo (ANID-Chile) through Ph.D. scholarship [ANID-PFCHA/Doctorado Nacional/2019-21190326], FONDECYT project [1180686], and CEDENNA [AFB180001]. Moreover, Dirección de Investigación científica y Tecnológica (DICYT-USACH) funded this publication.

## Conflict of Interest

The authors declare that the research was conducted in the absence of any commercial or financial relationships that could be construed as a potential conflict of interest.

## Publisher's Note

All claims expressed in this article are solely those of the authors and do not necessarily represent those of their affiliated organizations, or those of the publisher, the editors and the reviewers. Any product that may be evaluated in this article, or claim that may be made by its manufacturer, is not guaranteed or endorsed by the publisher.

## Abbreviations

AITC: allyl isothiocyanate
β-CD: beta-cyclodextrin
IC: inclusion complexes
RH: relative humidity
PM: physical mixture.
