# Supplementary material for: Development of Inclusion Complexes With Relative Humidity Responsive Capacity as Novel Antifungal Agents for Active Food Packaging
Source: Front Nutr. 2022 Jan 4;8:799779. doi: 10.3389/fnut.2021.799779 (PMC8764934; doi:10.3389/fnut.2021.799779)
Supplement: Supplementary file 1 [file Data_Sheet_1.docx]

Supplementary Material

# Field emission scanning electronic microscopy (FE-SEM)

The surface morphology of samples was investigated trough FE-SEM analysis. Samples were hold using carbon adhesive tabs and sputtered with a carbon layer. Images were obtained by using a Zeiss Merlin VP compact FE-SEM microscope (Jena, Germany) with a magnification of 150 x. Results are shown in Supplementary Figure 5.

# Water sorption isotherm of β-CD and β-CD:AITC inclusion complexes

Samples of 0.25 g of freeze dried β-CD, β-CD:AITC_2:1 and β-CD:AITC_1:1 were separately put inside desiccators with saturated salt solutions of potassium acetate (aw=0.23), magnesium nitrate (aw=0.50), sodium chloride (aw=0.75) and potassium sulfate (aw=0.97) at 20 °C. Water content was determined after 7 day-exposure and expressed in a dry basis (d.b.). Shape of curves was predicted using GraphPad Prism 7 software. Inclusion complexes were fit to sigmoid-type curve with R2 = 0.9994 for β-CD:AITC_2:1 and R2 = 0.9938 for β-CD:AITC_1:1. Results are represented in Supplementary Figure 7.

SUPPLEMENTARY FIGURE CAPTIONS

**Supplementary Figure 1.** Scheme of petri dish for antifungal assay.

Supplementary Figure 2. Scheme of AITC release from inclusion complexes to headspace at different relative humidity.

**Supplementary Figure 3.** Possible structures of β-CD:AITC inclusion complexes where isothiocyanate group is oriented to **a)** primary (SIST1) and **b)** secondary face of β-CD (SIST2).

**Supplementary Figure 4.** ^1^H-NMR spectrum of β-CD and β-CD:AITC inclusion complexes in DMSO-d_6_ obtained at 300 K.

**Supplementary Figure 5.** FE-SEM images of β-CD and β-CD:AITC_1:1 inclusion complex.

**Supplementary Figure 6.** Photo of antifungal assay against *B. cinerea* after 5 days from the remotion of inclusion complexes β-CD:AITC 1:1 (10 g) (day 10 of the assay).

**Figure Supplementary 7.** Sorption isotherms of pure β-CD and β-CD:AITC inclusion complexes at 20 °C. Values are mean of three replicates. Error bars lower than 3% of the absolute values lye below the symbols.
