# Supplementary figures and images for: Development of Inclusion Complexes With Relative Humidity Responsive Capacity as Novel Antifungal Agents for Active Food Packaging
Source: Front Nutr. 2022 Jan 4;8:799779. doi: 10.3389/fnut.2021.799779 (PMC8764934; doi:10.3389/fnut.2021.799779)

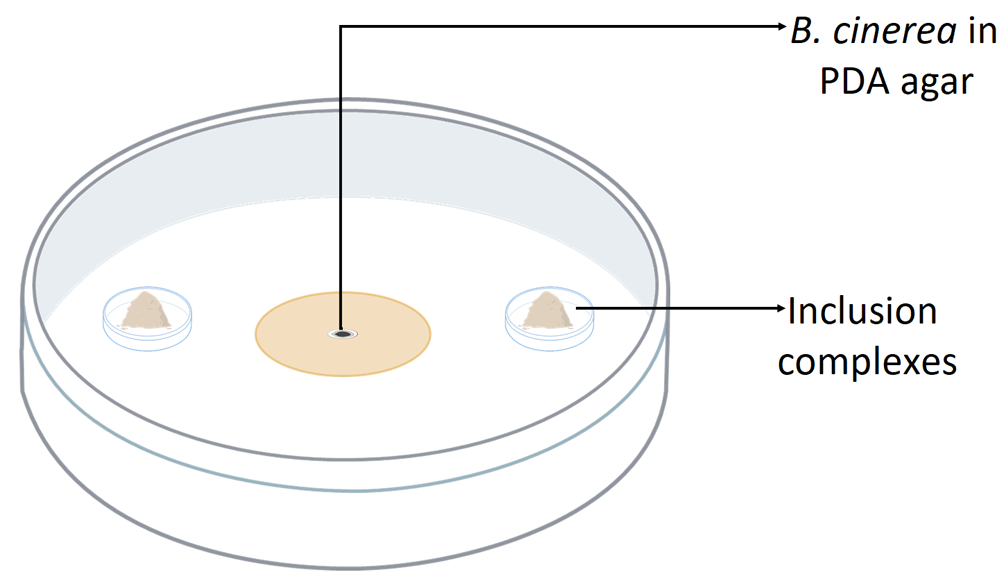

Supplement: Supplementary file 2 [file Image_1.TIF]

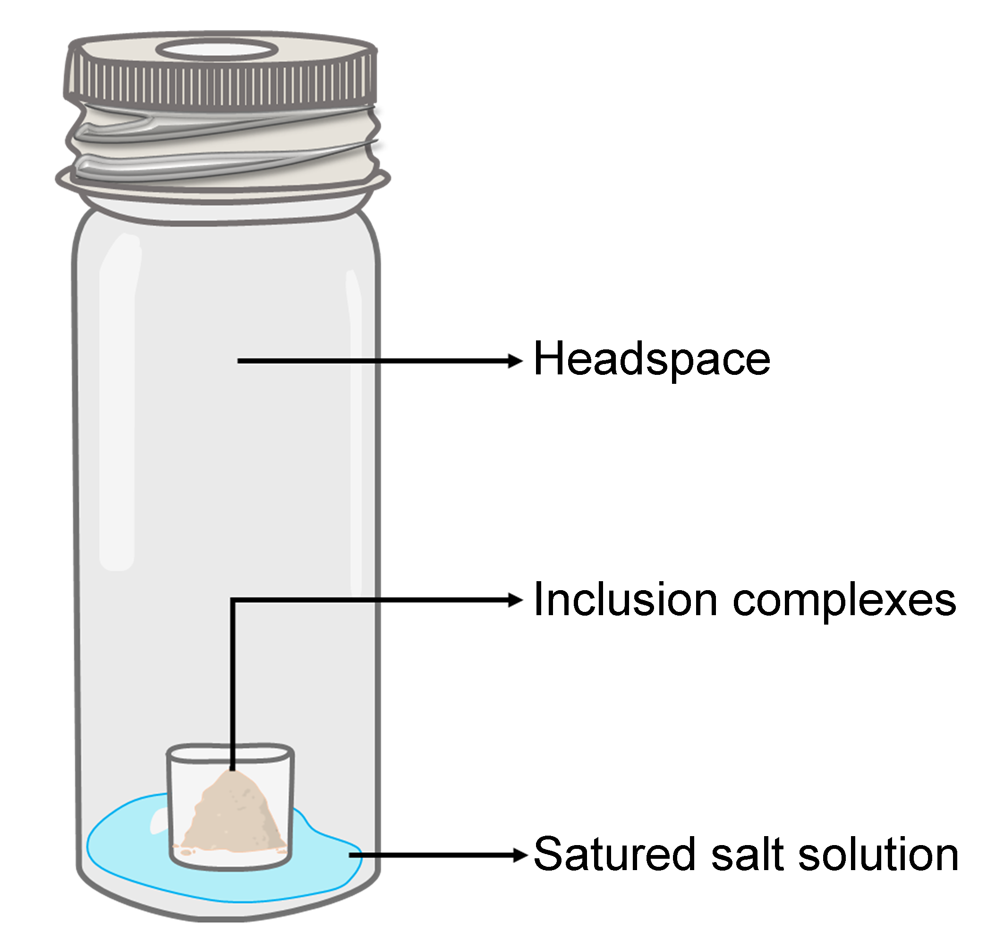

Supplement: Supplementary file 3 [file Image_2.TIF]

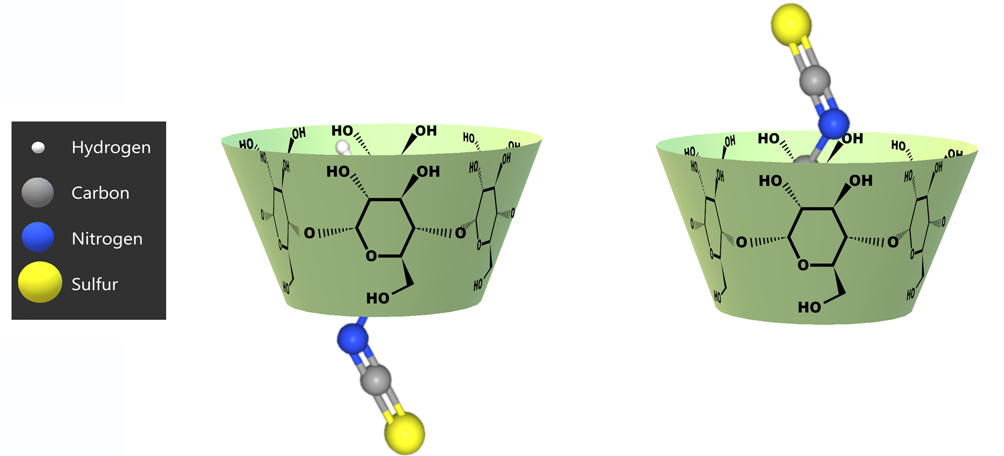

Supplement: Supplementary file 4 [file Image_3.TIF]

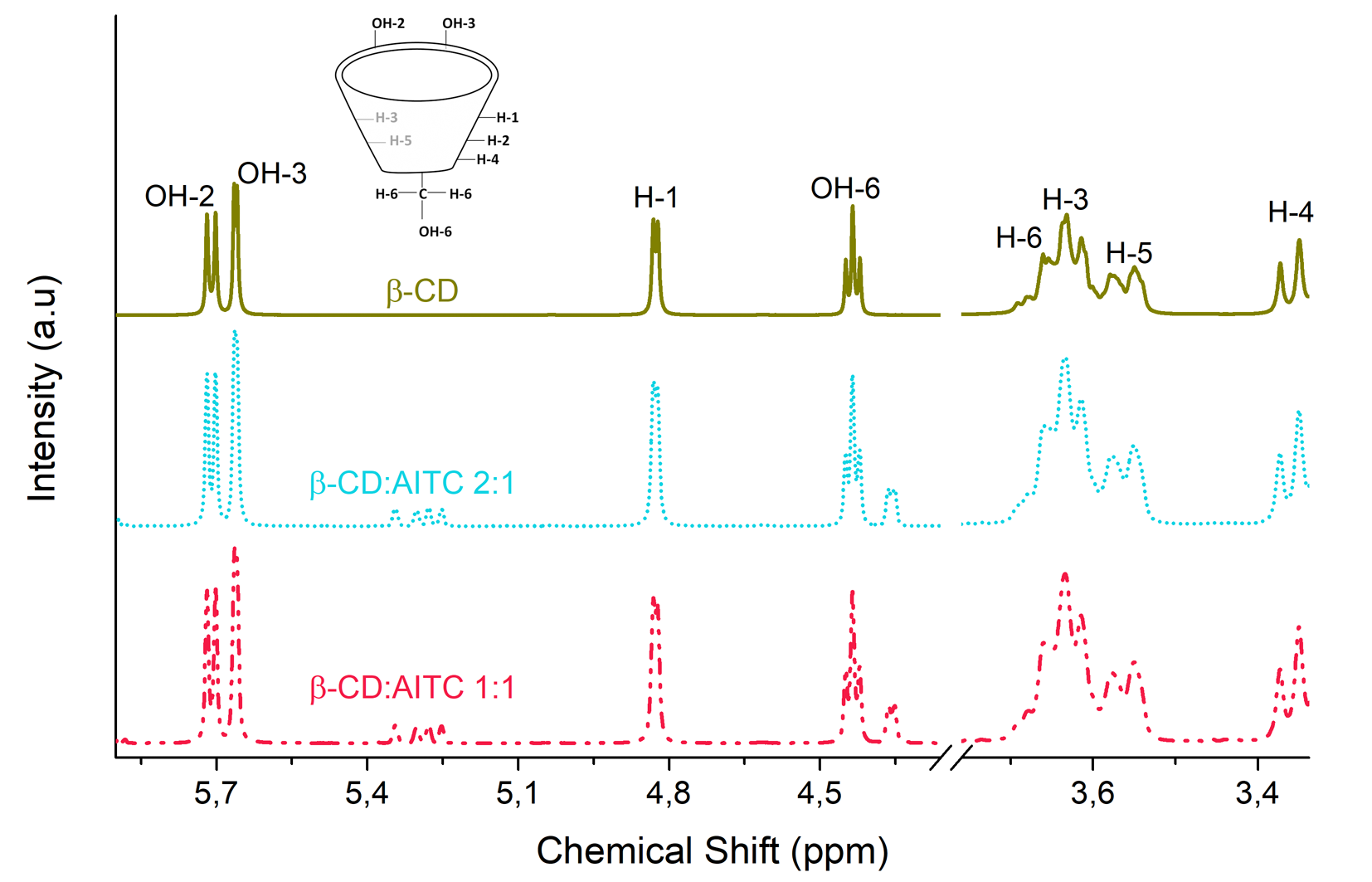

Supplement: Supplementary file 5 [file Image_4.TIF]

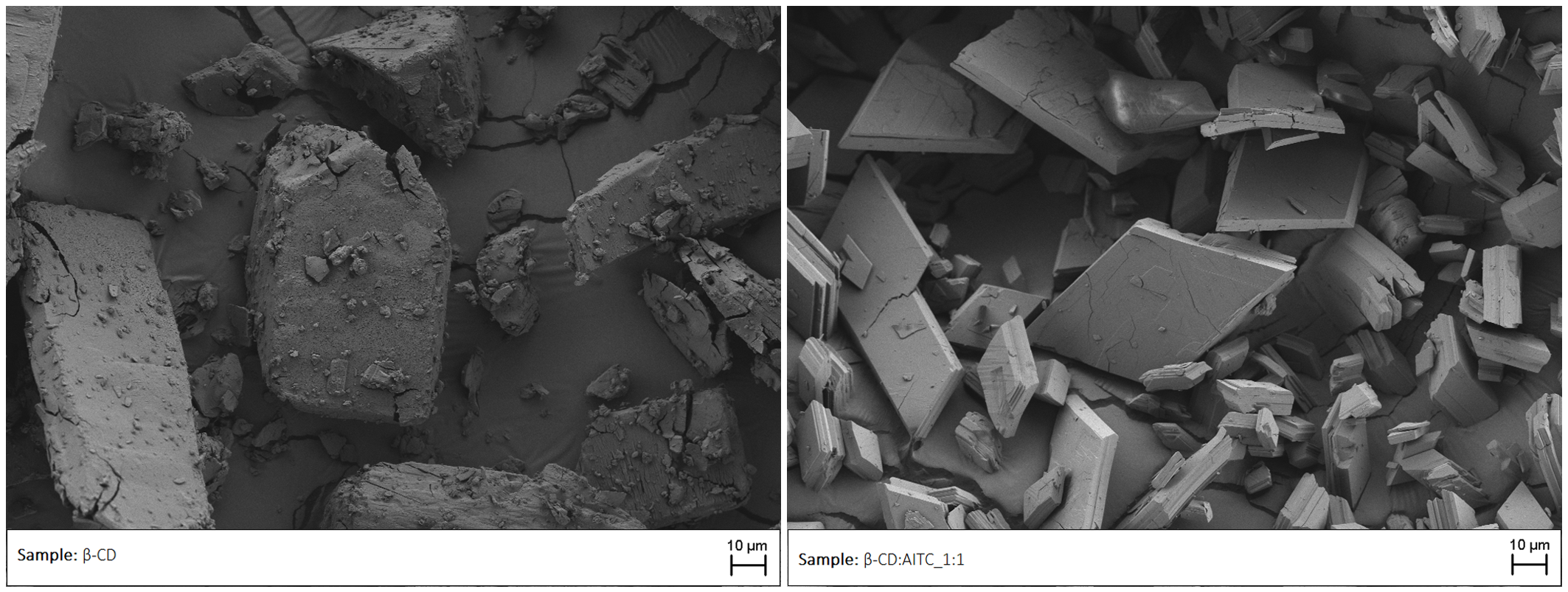

Supplement: Supplementary file 6 [file Image_5.TIF]

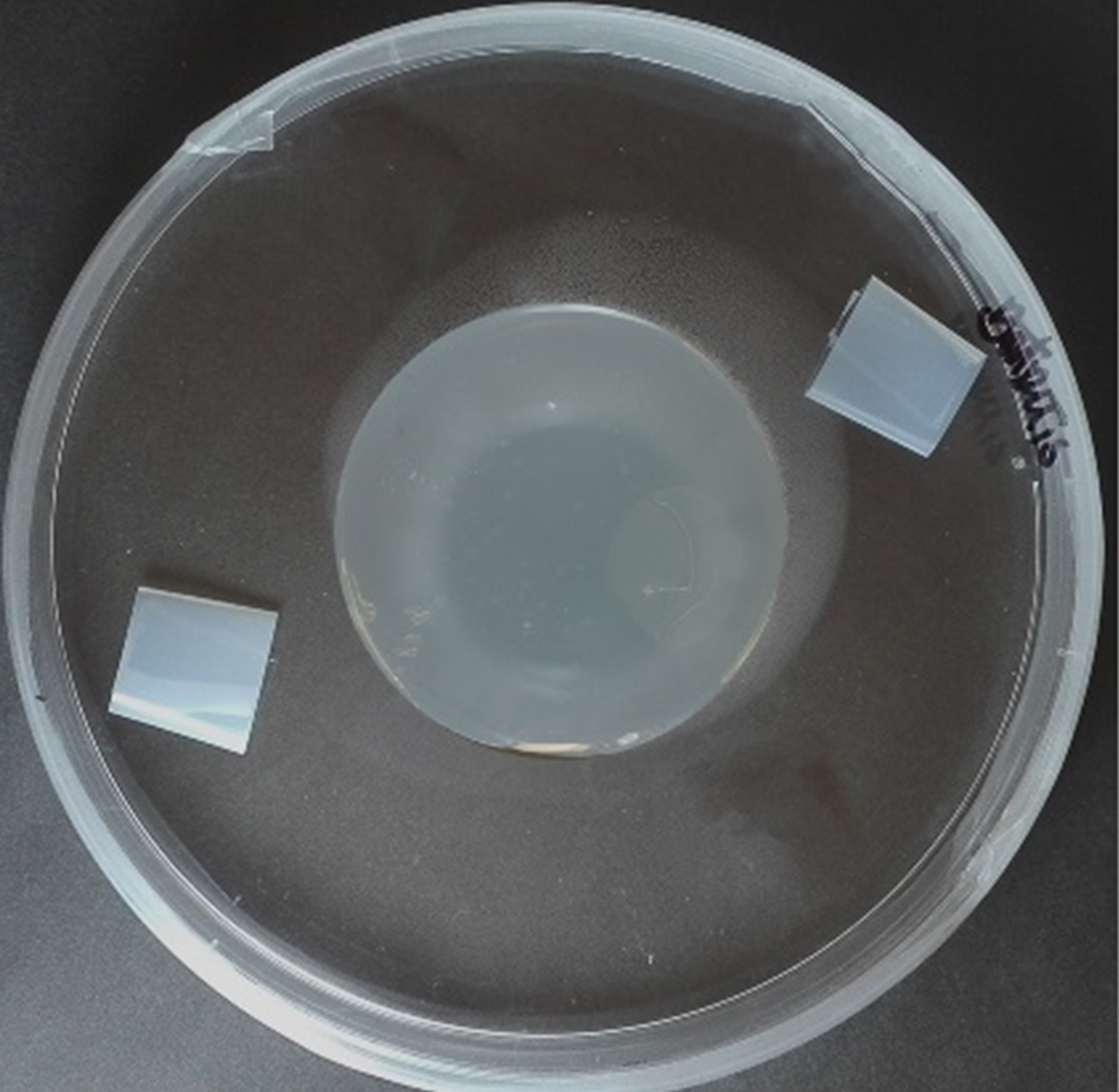

Supplement: Supplementary file 7 [file Image_6.TIF]

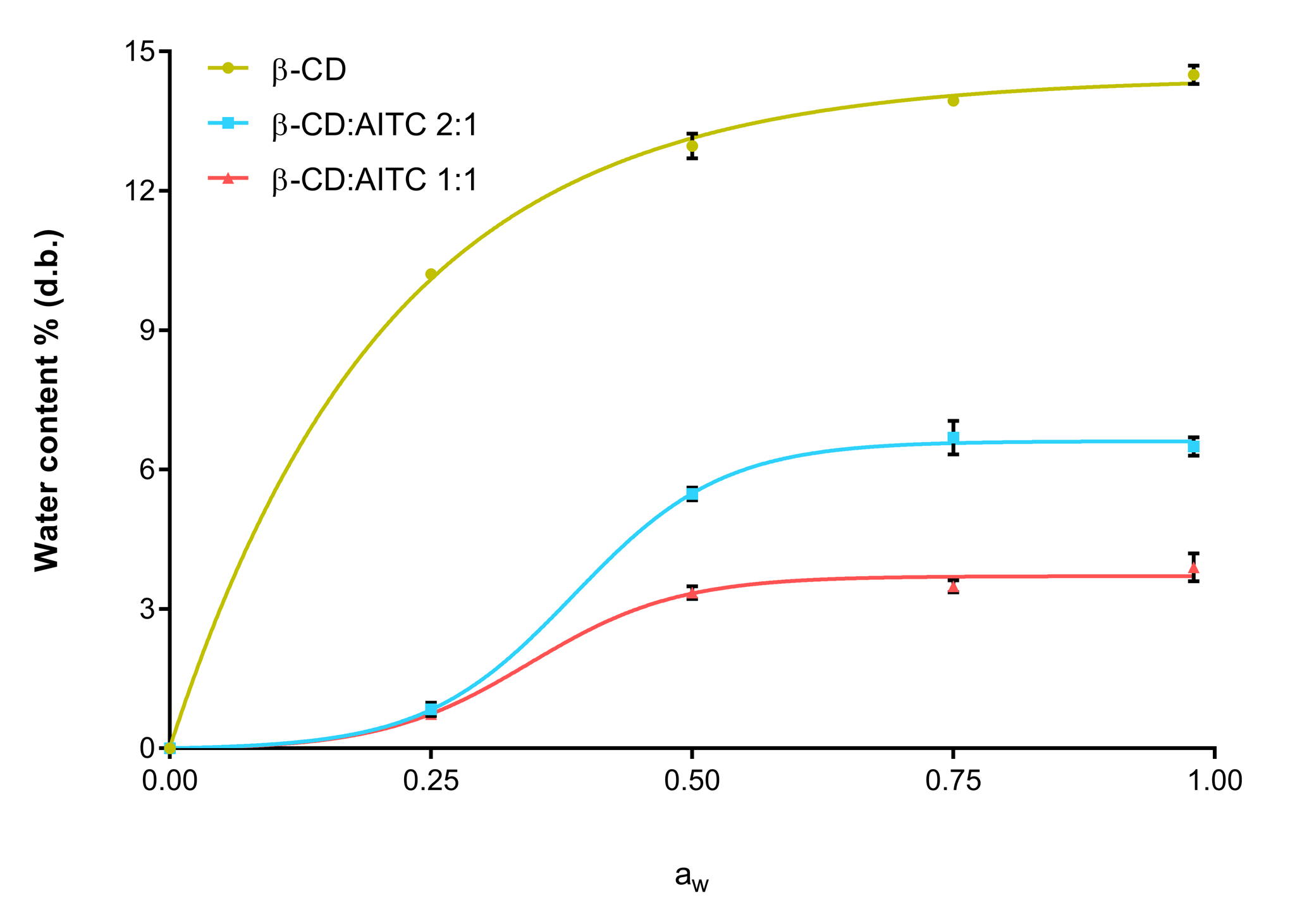

Supplement: Supplementary file 8 [file Image_7.TIF]
